# Expanding continuous glucose monitoring’s potential from real-time safety to lifestyle support in type 1 diabetes exercise: a proof-of-concept study among adults with low baseline exercise levels

**DOI:** 10.1016/j.hfh.2026.100154

**Published:** 2026-08-06

**Authors:** Young In Chung, Garrett I. Ash, Sangchoon Jeon, Reshma Ramachandran, Matthew Stults-Kolehmainen, Elias K Spanakis, Lisa M. Fucito

**Affiliations:** a Yale School of Medicine, Department of Psychiatry, New Haven, CT, USA; b Yale School of Medicine, Department of General Internal Medicine, New Haven, CT, USA; c Yale School of Nursing, New Haven, CT, USA; d Yale-New Haven Hospital, CT, USA; e University of Maryland, Baltimore Veterans Administrative Medical Center, Medicine and Endocrinology, MD, USA

**Keywords:** Continuous glucose monitoring, Type 1 diabetes, Exercise, Wearable electronic devices

## Abstract

**Objective:**

This proof-of-concept study sought to explore how data from CGM and exercise can be aggregated and analyzed to capture clinically meaningful glycemic patterns and actionable feedback. To this end, we examined how exercise days were associated with glucose outcomes derived from continuous glucose monitoring (CGM) among adults with type 1 diabetes (T1D) who had low baseline exercise levels.

**Methods:**

Secondary analyses were conducted on data from a 10-week digital app-based exercise intervention. Participants (N = 17; 52.9% Female; *M [SD]* age = 43.4 years [13.5]; *M [SD]* diabetes duration = 23.1 years [15.2]) received an exercise app with videos, text-based exercise coaching, a web-based self-monitoring diary, and a monthly session with personalized integrated feedback of CGM and other psychosocial data (exercise, mood, and sleep). To identify meaningful post-exercise periods, glucose patterns were examined across the 24-hour period, followed by iterative testing of different analytic windows. The final models focused on evening and overnight periods (18:00–06:00) to capture immediate and delayed glycemic effects. CGM-derived outcomes – mean glucose, time in range (TIR; 70–180 mg/dL), time in hyperglycemia (≥250 mg/dL), and time in hypoglycemia (≤70 mg/dL) – were analyzed using generalized linear mixed models. Logistic mixed models estimated odds of hyperglycemia and hypoglycemia, adjusting for age, diabetes duration, BMI, weekday, and HbA1c.

**Results:**

Relative to non-exercise days, exercise days were associated with lower mean glucose (−3.13 mg/dL, *p* = .001) and higher TIR (3.36%, *p* < .001), corresponding to ~25 additional minutes per night in range. Exercise days were associated with reduced time in hyperglycemia (−2.16%, p < .001) and lower odds of hyperglycemia (OR = 0.68 [0.58–0.78], *p* < .001). Exercise was associated with a small but statistically significant increase in percent time below 70 mg/dL (0.81%, *p* = .002); however, the interval-level odds model did not show a statistically significant increase in hypoglycemia risk (OR = 0.85 [0.71–1.00], *p* = .057).

**Conclusions:**

In this exploratory study, exercise was associated with greater evening and overnight glucose stability among adults with T1D who were physically inactive at baseline (i.e., 0 min of recorded exercise per week). These preliminary findings suggest the analytic feasibility of linking CGM and exercise data to identify clinically meaningful glycemic patterns. This lays the groundwork for an integrated behavioral support system that provides interpretable, actionable feedback to support patients’ diabetes self-management.

## Introduction

The use of continuous biometric data in the management of type 1 diabetes (T1D) has seen rapid growth, transforming the ways patients monitor their health and health care providers deliver care (Afridi et al., 2025). Continuous Glucose Monitoring (CGM) is at the forefront of this emerging trend by providing real-time glucose measurements from interstitial fluid. Unlike the traditional finger-prick tests that provide a single point-in-time measure, or the HbA1c levels (glycated hemoglobin) that reflect long-term glucose exposure (Cappon et al., 2019), CGM has the unique benefit of providing real-time, continuous glycemic patterns (Bergenstal et al., 2018). At its early stage of adoption, CGM was primarily issued as a medical safety device for those receiving intensive insulin therapy. Its function was largely automated and clinician-centered: sending warnings of imminent hypoglycemia, giving input to automated insulin devices, and informing endocrinologists on insulin adjustments (Battelino et al., 2019). However, this automation-centered design introduces a human factors concern: even the most advanced automated insulin delivery (AID) systems are not fully closed-loop and require ongoing human input to function safely. For example, most AID systems require patients to manually enter carbohydrate estimates before meals – a task prone to inaccuracy that can result in hypoglycemia or hyperglycemia. Acute conditions such as illness or glucocorticosteroid use can also cause rapid, transient glucose changes that fall outside the operating parameters of current algorithms, requiring patients to resume manual management without adequate preparation (Bainbridge, 1983). When automated systems fail or exit their operating boundaries under any of these conditions, the burden of managing the situation falls back on the patient or their caregivers. The growing integration of artificial intelligence and data-driven tools in healthcare underscores the importance of centering human factors principles such as usability, cognitive load, and patient-centered design in the development of these systems (Asan & Choudhury, 2021).

Beyond this primary use for medical safety, there has been a growing interest in leveraging the richness of CGM data to support measurable behavior changes in everyday life by giving users insights about their health that can guide their daily decisions. Daily management of T1D is demanding and often unpredictable, requiring individuals to constantly balance insulin dosing, dietary intake, physical activity, and stress. Such ongoing vigilance, along with the accompanying emotional toll, has been characterized as “diabetes distress” and contribute to chronic stress and diminished quality of life (Rodríguez-Muñoz et al., 2024; Skinner et al., 2020). Patients also struggle with anxiety about hyperglycemia (period of elevated blood glucose) and hypoglycemia (low blood glucose) which could lead to life-threatening complications if untreated. Although real-time CGM glucose values are undoubtedly beneficial for detecting these glycemic risks, CGM alone may not be sufficient for patients to navigate decisions around complex scenarios like exercise.

Exercise is a cornerstone of daily T1D management but is also one of the most challenging lifestyle behaviors to manage for patients. Evidence from large-scale studies such as the Type 1 Diabetes and Exercise Initiative (T1DEXI) (Riddell et al., 2023) shows that adults with T1D spent 6% more time in range (70–180 mg/dL) on exercise days compared to sedentary days. However, these benefits have been observed among individuals already accustomed to regular physical activity, and less is known about how these effects translate to adults who are new to exercise. Predicting glucose dynamics during and post-exercise is difficult due to its interplay with insulin pharmacodynamics and varying exercise intensity and duration. As a result, maintaining glucose stability post-exercise requires careful balancing of insulin and carbohydrate intake adjustments to prevent both hyperglycemia and hypoglycemia (Cockcroft et al., 2020) – a task that can be particularly challenging for those newly engaging in exercise (Brockman et al., 2021; Custy et al., 2024).

From a human factors perspective, the challenge of exercise management in T1D is not only physiological but also a problem of human cognition. Patients must perceive new information from multiple data streams, construct inferences about how exercise, insulin, sleep, and mood interact, and translate those inferences into safe daily action. This cycle is characterized as the core of sensemaking in diabetes self-management (Mamykina et al., 2015). When this sensemaking process is unsupported, individuals are left to interpret a continuous influx of data in isolation, leading to cognitive overload, frustration, and disengagement. Existing tools that present raw data without facilitating interpretation of patterns risk reducing self-monitoring to a ‘spectator activity’ – watching numbers go up and down without clear connection to action (Bond & Hewitt-Taylor, 2014). Designing feedback systems that support the perception-inference-action cycle is therefore a central human factors challenge in T1D self-management.

Considering these challenges, integrated feedback that links CGM data with exercise could help patients better understand these dynamics and make safer, more confident decisions in their daily life. Providing integrated feedback about the magnitude and time course of glucose changes following exercise can help alleviate concerns, form realistic expectations about glycemic outcomes after exercise, and boost self-efficacy. Moreover, sustainable health-promoting behavior change requires not only initial motivation but also ongoing reinforcement (Kwasnicka et al., 2016; Michaelsen & Esch, 2023). For those who lack access to more intensive, sustained human coaching for exercise, biometric feedback from CGM could serve as a personalized, data-driven reinforcement system to enhance internal motivation. It could also help maintain motivation in between human-delivered exercise coaching sessions, enabling patients to achieve greater consistency in their exercise adherence.

Despite technological advances in CGM, questions remain about how best to analyze and integrate CGM with other behavioral data streams such as exercise in ways that are safe, interpretable, and actionable. Consensus summary metrics (i.e., the Standard Ambulatory Glucose Profile) (Battelino et al., 2023; Bergenstal et al., 2013) and machine-learning based pattern detection algorithms (Woldaregay et al., 2019; Zhu et al., 2022) offer partial solutions, but no established framework (i.e., across which CGM metrics and time window) yet connects CGM readings to everyday behaviors like exercise in a patient-centered way. A first step toward this goal is to characterize the CGM-exercise relationship at the group level using standardized metrics and varying time windows, providing an evidence base that can inform personalized feedback.

Therefore, this proof-of-concept study sought to explore how data from CGM and exercise can be aggregated and analyzed to capture clinically meaningful post-exercise effects. This secondary analysis of a 10-week pilot exercise intervention examined associations between exercise and glucose trends among adults with T1D who were beginning to engage in exercise and explored associations across multiple CGM consensus metrics and time windows. Glucose patterns were first explored across the 24-hour period, followed by iterative testing of different post-exercise windows to capture the delayed glycemic effects. In particular, the analysis focused on evening and overnight glucose trends (18:00–6:00 the following day) to assess post-exercise glucose stability and temporal changes in hyper-and hypoglycemia risk. The evening and overnight window is a clinically important timeframe to monitor as the delayed effects of exercise on insulin sensitivity can increase nocturnal hypoglycemia risk (Basu et al., 2014; Valli et al., 2021), which is a major barrier for individuals with T1D to engage in exercise (Brazeau et al., 2008). Therefore, when starting or changing one’s exercise routine, it is important to understand the glucose dynamics during this “exercise risk window.” By identifying interpretable, fine-grained glucose trends on exercise days, this work aims to test the analytic feasibility and inform the future design of integrated biometric feedback systems that could support patient-led diabetes self-management.

## Problem statement

Despite the widespread use of CGM in T1D management, its potential to support daily behavioral decisions around exercise remains underutilized. This study addresses the gap by examining how CGM and exercise data can be aggregated and analyzed across multiple CGM metrics and time windows to characterize the magnitude and temporal changes in glucose levels and glycemic risks on exercise days relative to no-exercise days. These findings provide foundational evidence for developing integrated, clinically meaningful CGM feedback systems.

## Methods

A detailed description of the study design, recruitment procedures, intervention components, and assessments has been published previously (Ash et al., 2023). In brief, adults aged 18–65 years with ≥6 months’ diagnosis of type 1 diabetes or other absolute insulin deficiency diabetes, and low baseline exercise (<3 sessions/week) were recruited to participate in the study. Participants received a 10-week intervention that combined a smartphone application offering exercise video and support, text-based exercise coaching, and monthly session with personalized CGM feedback. All participants were current users of CGM and owned smartphones. Exclusion criteria included chronic illness or physical limitations that require exercise modifications outside the scope of the study’s intervention. The study was approved by the Yale University Institutional Review Board, and all participants provided written informed consent.

### Study design

In a single-group design, participants completed a two-week baseline followed by a 10-week intervention period. Clinical and psychosocial variables were assessed and observations (CGM, automated insulin delivery logging, exercise, and hip accelerometry) were recorded pre-and post-interventions. The intervention consisted of 1) access to a mobile application (GlucoseZone) with on-demand exercise videos and feedback on health outcomes, 2) text-based coaching and advice on exercise through the app when needed, 3) self-monitoring of exercise, sleep and mood states using web-based daily diary each morning, and 4) monthly motivational enhancement therapy sessions with a counselor, incorporating feedback from biosensors (CGM, Apple watch, and self-monitoring).

All exercise videos were from GlucoseZone’s publicly available version (GlucoseZone.com; Fitscript LLC, New Haven, CT), with each video ranging from 20 to 50 min in length. Participants could customize their exercise by selecting the difficulty level, duration, type of exercise (aerobic, resistance, flexibility and balance), muscle groups, and mobility limitations (knee-friendly, shoulder-friendly, chair-based).

Each monthly session was conducted by the principal investigator (G.I.A., trained in motivational enhancement therapy by a clinical psychologist, L.M.F.). A structured visual report presenting personalized biosensor data was used to guide these sessions. The report included exercise summary statistics, CGM-derived glucose summary statistics, benchmarked against clinical targets, and the intersection of CGM data with exercise, mood, and sleep variables side-by-side, enabling participants to observe how their glucose patterns varied on exercise versus non-exercise days (see Fig. 1 for an illustrative schematic of the report). The report was reviewed collaboratively, inviting participants’ reflections. Conversations also focused on exercise goals and barriers in a nonconfrontational style of motivational enhancement therapy. The objective of these sessions was to reduce the complexity of multi-signal sensemaking by presenting integrated, contextualized patterns to support patient-led decision-making between sessions.

### CGM data and measures for exercise

24-hour blood glucose levels were recorded by the participants’ personal CGM devices (Dexcom G6 (40%), G5 (10%), G4 (5%), Guardian (35%), and Abbott Freestyle Libre (10%)). Glucose levels were recorded every 5 min by the CGM devices (every 15 min for the Abbott Freestyle Libre). Each day, 24 hr summary metrics were recorded following the consensus CGM cut-points: mean glucose, variability (coefficient of variation), % of time in hyperglycemia (glucose over 180 mg/dL), % of time in clinical target range (70–180 mg/dL), and % of time in hypoglycemia (under 70 mg/dL) (Battelino et al., 2023).

Exercise was tracked by the on-demand exercise application usage timestamp log as well as participants’ self-monitoring of exercise using web-based daily diaries. Participants also wore an Apple Watch 3 (Cupertino, CA) to capture heart rate. Heart rate ≥50% age-predicted maximum while viewing the in-app exercise video was cross-checked to confirm exercise. Exercise days were defined as days in which any exercise was recorded.

### Analysis

We used generalized linear mixed models (GLMMs) to estimate the associations between exercise and CGM outcomes at the hourly and 5-minute levels. The 5-minute interval corresponds to the native CGM sampling frequency. We also examined the hourly averages to detect clinically interpretable glucose risk patterns. Following the international consensus guideline, CGM-derived outcome variables included mean glucose (mg/dL), percent of glucose values in the target time in range (TIR; 70–180 mg/dL), percent of values above 250 mg/dL (corresponding to level-2 hyperglycemia), and percent of values below 70 mg/dL (corresponding to level-1 hypoglycemia) (Battelino et al., 2023). Random intercepts were included to account for the correlation of within-person, repeated measurements. Independent variables included exercise (exercise day versus non-exercise day) or the post-exercise period (within 30 mins, 30 mins to 1 hrs, 1hr to 2 hrs, and later than 2 hrs) after adjusting for the demographic covariates.

To select post-exercise timeframe, we initially examined glucose patterns across the full 24-hour period. Prior studies have reported that the extended effects of exercise on glucose last anywhere around 2 to 48 h post-exercise (Basu et al., 2014; MacDonald, 1987; Sandoval et al., 2006). However, the physiological duration of exercise effects is not well established, particularly in our study population of previously inactive adults with T1D. Therefore, our analytic approach involved iteratively testing different time windows to capture the immediate and delayed glycemic effects. No significant differences were observed during daytime hours across the CGM derived metrics. Given the high density of within-person observations in this dataset, these null findings are consistent with an absence of detectable daytime effects in this sample. Based on these findings, the final analysis was restricted to the overnight window (18:00 through 06:00 the following day).

In addition, to examine exercise’s impact on glycemic risk, logistic regression models were used to estimate the odds of hyperglycemia (≥250 mg/dL, per 5-minute interval) and hypoglycemia (≤70 mg/dL, per 5-minute interval) for each 5-minute interval. Models were stratified by time since exercise (within 30 min, 30 min-1 hour, 1–2 h, and later than 2 h). Time of day, weekday, age, diabetes duration, and body mass index were included as covariates in each model. HbA1c (glycated hemoglobin) was also included as a covariate to account for between-person differences in baseline glucose regulation that could influence CGM-derived outcomes.

### Qualitative analysis of exit interview

At the end of the intervention period, participants completed a 30-minute individual, semi-structured exit interviews to elicit participants’ experiences with the intervention and its perceived impact on exercise and diabetes self-management. Interviews were conducted using secure videoconferencing or telephone by trained research staff, audio-recorded with consent, transcribed verbatim, and de-identified before analysis. Inductive thematic analysis with a qualitative interpretivist approach was used to identify key themes. An initial codebook was derived from a subset of transcripts, expanded iteratively as new themes emerged, and refined through consensus review with collaborators. A full qualitative analysis of the interview data is currently in preparation. In the present paper, we report on two themes directly relevant to the cognitive objectives of the integrated feedback intervention: sensemaking and motivation.

## Results

### Participants and intervention completion

Twenty participants enrolled but three participants were excluded due to insulin pump download error, manual insulin logging with unreasonable timestamps. For the analysis, seventeen participants (52.9% Female) with type 1 diabetes (90%) or latent autoimmune diabetes (10%) completed all outcomes measures. Participants were 43.4 ± 13.5 years old on average, with longstanding diabetes diagnosis (23.1 ± 15.2 years), BMI above the clinically recommended range, and HbA1c above the clinically recommended range at baseline (see Table 1 for demographics and clinical characteristics).

The dataset included a total of 1020 days of observation (41.3% exercise days and 58.7% non-exercise days across participants). 165 (14%) of the person-days were excluded due to missing survey or CGM data. Adherence of biosensor protocol was high ranging between 88% and 91% of days where biosensor data was collected. Specifically, for CGM data completion, participants contributed an average of 90% (SD = 9%) of expected CGM observations. Participants’ logged exercise duration increased from a median of 0 min/week at baseline to 64 min/week at the last 2 weeks of the intervention. 99% of the bouts of exercise amounted to ≥30 metabolic equivalent (MET)-minutes. 91% (SD = 14) of self-monitoring daily diary was completed. 85% of participants attended both monthly feedback sessions.

### Glucose profile and risk of hyper- and hypoglycemia on exercise days

Comparing each participant’s exercise days to their non-exercise days, days with exercise regardless of when exercise was conducted that day was significantly associated with several consensus summary metrics compared to their non-exercise days. Specifically, mean glucose was lower on exercise days vs. non exercise days (−3.13 mg/dL, *p* = .001). Likewise, time in target glucose range (70–180 mg/dL) was 3.36% higher on exercise than non-exercise days (*p* < .0001), which corresponds to an additional 24.5 min over the 12-hour window compared to non-exercise days. Exercise days were associated with 16 min reduced time per night in level-2 hyperglycemia (−2.16%, *p* < .0001) and 32% lower odds of hyperglycemia compared to non-exercise days (OR = 0.68 [0.58–0.78], *p* < .0001). Independent of exercise, the risk of hyperglycemia was highest on Fridays and Saturdays. For hypoglycemia, there was a slight increase in time (i.e., 6 min more per night) (0.81%, *p* = .002) on days with exercise as well as a nonsignificant trend toward lower odds of hypoglycemia (OR = 0.85 [0.71–1.00], *p* = .057) compared to non-exercise days. Risk of hypoglycemia was highest on Thursdays through Saturdays, independent of exercise.

Among covariates, HbA1c, as clinically expected, was closely linked with glucose metrics: higher HbA1c was associated with higher mean glucose, less time in range, more time in level-2 hyperglycemia, and higher odds of level-2 hyperglycemia. Main effects of other covariates (age, BMI, and diabetes duration) were not statistically significant. Results are summarized in Table 2-a and 2-b.

### Temporal changes in glucose and hyper- and hypoglycemia risk post-exercise

Post-exercise effect was most pronounced in the 1–2hr window after exercise (−4.84 mg/dL, *p* < .0001). The average glucose was lower within 30 min after exercise, compared to >2 h post-exercise, and this glucose-lowering effect continued to increase overtime. Consistent with mean glucose changes, the odds of hyperglycemia progressively decreased in the hours after exercise, with the greatest reduction in odds (43% lower compared to >2 h post-exercise) observed in the 1–2 hour window. The interval-level logistic model did not show a statistically significant increase in odds of hypoglycemia across post-exercise period. This should be interpreted alongside the daily-level finding that percent time below 70 mg/dL was slightly but significantly elevated on exercise days overall.

Within-person weekend effects were again observed. Regardless of exercise status, glucose levels were significantly higher on Fridays (5.52 mg/dL) and Saturdays (7.2 mg/dL) compared to Mondays. Both hyper- and hypoglycemia risks were elevated on Thursdays, Fridays, and Saturdays. Results are shown in Tables 3 and 4.

### Participant feedback on integrated feedback

In post-intervention acceptability interviews, participants provided qualitative feedback about their experience reviewing monthly integrated CGM-exercise feedback with a counselor. Participants reported that these discussions about exercise and its intersection with biosensor data were among the most satisfying components of the study. Two themes emerged from the interview data relevant to the cognitive objectives of the integrated feedback intervention: sensemaking and motivation.

Sensemaking: A prominent theme was the cognitive challenge of managing and interpreting information from multiple separate data streams, and the value of the monthly integrated feedback sessions in reducing that burden. Participants described their everyday experience of juggling multiple devices and data sources as overwhelming. One participant stated, “*blood-sugar meter in the other hand, and making sure the watch is syncing, and I have my other phone on me, so I [am kind of] like whatever, and I’m just like “aah!”, like it’s so much…*” (Participant 1). In contrast, participants found that the monthly integrated report made complex, multi-stream data concise and interpretable. One participant contrasted the integrated report with their existing CGM platform, *“[CareLink] is …. very clunky, in the sense that, there’s a ton of data in there, but it’s very difficult to navigate through all that data….The monthly report goes into more detail, because the live reports, aren’t able to show all CGM data, so the report on, GlucoseZone, only shows for that hour to not, what can happen after or before as a result of the, exercise. Whereas, the monthly reports are able to, show that information*” (Participant 19). Another participant described the integrated feedback as informative in addition to their CGM data in isolation: “*I think it [often] comes back to the, to the, to the monthly meetings with you to go through the numbers, I think I found that more interesting than anything, seeing all of the data in front of you, because I’m always seeing my Dexcom data but not seeing Dexcom data layered in with sleep and exercise, so nicely done*” (Participant 23). Another reflected on how the integration supported perception of data: *“I’ve, have, 35 years of doctors’ appointments under my belt where, where people tell me like “you know, you gotta do more exercise [and eat], coun-, watch your carbs”, all that stuff. This is like the first time that I really get to see, I get to see something tangible behind that, that showed what happens when you do it and what happens when you don’t. And, I think it’s clear that, the body changes when you do exercise and it, helps out a lot. I’m, I’ve always been more of a visual person, and I think, seeing that has really, has like changed things a little bit for me”*(Participant 21). The participant also described a distinction between sensemaking and habitual modes of self-management and that the intervention helped them develop curiosity and inferences from their own data: “*the study got me in the habit of wearing that, and then, looking at more in-depth with it, than I had before at all. I, didn’t even really know that there were this many, options there, that I can go in and see like the, the, the patterns and everything*” (Participant 21). Another participant commented about how they began independently applying the insights from the integrated report outside of counselor sessions: “*I understood the theory in general … because I had, read about it, but I hadn’t really noticed it too much within myself, besides a little bit last summer, I would sometimes notice like, if I, you know, we’re going for a walk or a hike, … I learned not to, not to dose insulin, when I was exercising, unless I was, well out of range to begin with but, yeah, I learned that lesson*” (Participant 1).

Motivation: A second theme was that reviewing quantitative data about their own exercise and glycemic patterns was experienced as reinforcing and motivating. One participant commented about being motivated by seeing evidence of their progress quantitatively: *“I really liked the follow-up and review, I thought that was fantastic, I love seeing all of the stats… how did this go? What changes were made? Do you find that there was extra effort that you put in, did it pay off, did it not?… I am highly motivated by seeing progress numbers, where can I make an improvement, that was awesome”* (Participant 12). Another participant noted “*what kept me going was really just, those monthly sessions and trying to improve, every month*” (Participant 19).

## Discussion

This study illustrates how CGM and exercise data can be leveraged as a use case to support exercise among adults with T1D. To achieve this goal, we conducted a series of analyses to demonstrate how patterns of glucose stability and the timing and magnitude of glycemic risk can be derived from high-resolution CGM data. We identified preliminary associations that could be useful for guiding safe and effective exercise engagement. Exercise days were associated with lower average glucose and greater overnight stability. While a small but significant increase in percent time below 70 mg/dL was observed, the odds-ratio model did not show a significant increase in hypoglycemia risk during the clinically relevant evening and overnight period in adults with T1D who are new to regular exercise. Importantly, no significant effects of exercise were observed during daytime hours, reinforcing the clinical relevance of the overnight “exercise risk window,” during which glucose stabilization are most critical. On exercise days, participants spent less time in hyperglycemia range, and exercise days were associated with lower odds of hyperglycemia, approximately one-third compared to non-exercise days – a potentially meaningful reduction from a quality-of-life standpoint given the consistent fear of hyperglycemic events in this population (Singh et al., 2014). While the absolute change in mean glucose was modest, the significant reduction in hyperglycemia without evidence of increased hypoglycemic risk in the interval-level odds model suggests the potential of integrated CGM and exercise data to capture clinically relevant patterns. It should be noted that although the dense repeated-measures structure of the data improves within-person precision for estimating these odds rations, this does not compensate for the small number of participants (N = 17) or establish population-level stability of these effect sizes. The observed Ors should therefore be interpreted as preliminary within sample estimates. From a proof-of-concept perspective, these findings highlight how real-world biometric data when contextualized with behavioral information, can be leveraged to support lifestyle changes.

The current findings align with the results from a large-scale T1DEXI study (Riddell et al., 2023) which focused on adults with established exercise habits. A consistent but smaller reduction in average glucose level was observed (e.g., mean glucose decline 3.13 mg/dL compared to T1DEXI that ranges from 9–18 mg/dL depending on the exercise type; 3.36% increase in TIR over 12 h compared to 6% increase in TIR over 24 h in T1DEXI), highlighting that measurable benefits can still emerge early in exercise engagement. Consistent with recent evidence that showed that higher daily step count and moderate activity increased time-in-range and reduced insulin needs (Pemberton et al., 2025; Turner et al., 2024), these results underscore the sensitivity of CGM metrics for capturing even brief physical activity. In addition, weekend elevations in hyper- and hypoglycemia risk replicated known within-person fluctuation in glucose and metabolic outcomes (e.g., Clemmensen et al., 2022 on weekday variation in glucose/insulin; Belsare et al., 2023 on seasonal CGM trends). Lifestyle factors such as diet and sleep have been studied as mediators of the within-person glucose variability (Griggs et al., 2020). Using these integrated feedback, providers could explicitly compare weekday and weekend patterns with patients and discuss strategies to plan and sustain self-management of health behaviors during higher-risk days. In addition, use of time-sensitive messaging and just-in-time intervention such as targeted prompts on weekends, may be designed around these susceptible periods. As clinically expected and consistent with prior work, HbA1c emerged as a key between-person predictor of overall glucose metrics (Eliasson et al., 2024). This finding highlights the importance of personalized feedback for patients as the magnitude of exercise effect (i.e., 24.5 more minutes in range) may be perceived differently depending on the patients’ baseline HbA1c level. Visualization of the associations between exercise and glucose level should consider accounting for baseline HbA1c so that patients across diverse glycemic profiles could see the benefits of exercising and perceive their personal progress over time.

From a human factors perspective, this study demonstrates how CGM and exercise data can be integrated to generate interpretable feedback for self-management. While wearables and digital health applications often present data from individual streams separately (e.g., glucose readings, step counts, sleep patterns), there is a greater need for integrated feedback. Linking CGM patterns directly to exercise could help make information from multiple data streams more tangible, reassuring, and immediately rewarding for users, helping reinforce exercise behaviors. The time course of the glucose-stabilizing effect of exercise in the evening and overnight could offer important opportunities for actionable feedback as well. For example, a user who exercises in the afternoon could receive a personalized summary showing that their overnight glucose was more stable on exercise days, with fewer excursions above 250 mg/dL, translating a complex, multi-day CGM pattern into a motivating insight. This kind of feedback could directly support planning post-exercise bedtime routines around anticipated glucose trends, adjusting the timing or intensity of exercise to optimize overnight stability, or calibrating carbohydrate intake around the exercise risk window. These cross-stream feedback systems showing the connection between users’ behaviors and glucose outcomes could simplify self-management (rather than monitoring multiple dashboards in isolation) and reinforce behavior changes. The principles of making high-resolution biometric data more personally relevant and interpretable by combining it with behavioral data and contextual time windows go beyond health technologies for diabetes. The analytic approach used here is applicable to the designing of digital health systems across chronic disease management (Tandon et al., 2024).

Consistent with the sensemaking framework for chronic disease self-management (Mamykina et al., 2015), the qualitative findings provide participant-level warrant that the integrated feedback intervention supported key cognitive objectives relevant to T1D self-management. At the perception stage, our integrated feedback approach provided a visual report surfacing CGM-exercise patterns that participants had not been able to observe in their existing platforms. At the inference stage, the intervention facilitated active construction of explanations linking exercise behavior to glycemic outcomes. Participants described developing new curiosity and analytic engagement with their own data, consistent with the shift from habitual to active sensemaking mode. At the action stage, participants described developing practical exercise management skills and motivation to improve month over month based on what they observed in the data. Importantly, there was qualitative evidence that some participants began applying these insights independently, outside of the monthly counselor sessions – for example, learning not to dose insulin during exercise unless substantially out of range. This suggests that the integrated feedback approach began to support the kind of autonomous sensemaking and self-directed action that would be necessary for independent long-term self-management. These qualitative findings are consistent with the interpretation that the cognitive design objective contributed to the observed clinical outcomes, though causal conclusions await further investigation.

This study has several limitations that should be taken into account when considering its generalizability. First, while the repeated-measures structure of the generalized linear mixed and logistic models provides stable within-person precision for the reported odds ratios, between-person generalizability remains an important constraint. With 17 individuals, it is possible that the observed effect sizes reflect characteristics specific to this sample rather than the broader population of adults with T1D. The sample’s demographic homogeneity (e.g., non-Hispanic White, technology-familiar) further limits generalizability. These findings should therefore be interpreted as preliminary signals motivating larger, more diverse confirmatory studies rather than as definitive estimates of the CGM-exercise relationship. Replication in larger samples is needed before these effect sizes can be considered reliable. Second, behavioral factors known to affect glucose variability such as insulin administration, dietary intake, stress, and sleep patterns, were not accounted for in these analyses. However, even without isolating different physiological mechanisms, the significant reduction in hyperglycemia suggests that linking exercise data with CGM could help individuals identify practical strategies for exercise. Third, exercise was defined by app usage logs and self-report diary entries, which may not capture all physical activity outside the app. Fourth, we did not look at the differences in intensity, duration, or type of exercise in our analyses which may influence glucose response (Riddell et al., 2023). Given the small sample and limited variability in exercise routines, the present study focused on characterizing the generalizable patterns of glycemic change on exercise days. Future work should explore how variations in exercise type and amount contribute to glucose responses. Finally, CGM devices varied across participants, which may introduce measurement variability in glucose outcomes.

Despite these limitations, the study has several notable strengths. Data completion rate was high with participants providing on average 90% of expected CGM recordings across >1000 observations days that included a balanced mix of exercise and non-exercise days. This increases validity and robustness to the observed patterns. It is also worth noting that adherence to the exercise videos was high, with a very low rate of early abandonment. Although exercise was coded as a binary variable rather than a continuous measure, the high adherence rate reduces the concern that “exercise days” represented only minimal activity. Moreover, our backend monitoring of app usage provided objective confirmation of exercise engagement – a level of verification that is standard in research settings but generally absent from commercial platforms such as Dexcom Clarity or Abbott LibreView, which typically rely on patient self-report of exercise. This high level of monitoring enhances confidence that the observed associations reflect genuine behavioral-physiological relationships. By integrating a commercially accessible, behavior-change–oriented platform (Glucose-Zone) with research-grade data collection methods, this study demonstrates the feasibility of combining real-world usability with analytic rigor in future digital health research. Looking ahead, if the analytic approach demonstrated here were adapted for real-time delivery, these patterns could be available to individual users rather than requiring group-level analysis, representing a key next step toward a biosensor-based behavioral support system for T1D.

Future work should focus on designing and testing user-centered biometric feedback systems that integrate information from multiple data streams in a way that is clear and actionable, without adding to the diabetes distress with technological burden and information overload (Fagherazzi, 2023). Real-time self-monitoring of multiple, separate data streams for patients is a useful feature (Winterdijk et al., 2023), but there is a clear opportunity for integrated feedback, such as the exercise and CGM relationship. Realizing this potential will require careful attention to visualization design that reduces cognitive complexity. Future integrated feedback systems could present simple, within-person summaries first – for example, showing the user that their overnight glucose was more stable on exercise days than non-exercise days in a single graphic. Then, it could offer optional drill-down into finer-grained patterns such as hourly glucose trajectories or day-of-week variations. Visualization anchored to within-person change, rather than population norms, would allow users across diverse glycemic profiles to perceive their own progress. In addition, one key factor to evaluate is how varying frequencies of such integrated feedback could enhance exercise engagement without leading to information overload. A subsequent trial from this research team found that providing feedback weekly, rather than monthly, increased participant satisfaction, suggesting that further testing the optimal frequency of feedback is warranted (Ash et al., 2025). One important caveat to consider is that user preferences are likely to vary (Cho et al., 2022). Therefore, giving users opportunities to tailor how often they interact with these health apps may be a promising strategy to enhance both satisfaction and engagement. Individuals living with T1D already manage many aspects of their condition on a daily basis; therefore, when new technologies are introduced, it is especially important that they are easy to use and customize, and are preferably consolidated into a single, streamlined system to reduce redundancy.

## Conclusion

This proof-of-concept analysis suggests that CGM data can be used to detect preliminary associations between exercise and glycemic outcomes in adults with T1D. Exercise days were characterized by lower mean glucose and reduced hyperglycemia risk during the clinically important evening and overnight period, without evidence of a substantial increase in hypoglycemia risk in the interval-level odds model. While these findings require replication in larger confirmatory studies, they suggest that such patterns could be integrated into a CGM-based feedback system, presenting users with personalized and contextualized information that could help enhance exercise motivation and support long-term behavioral changes. In order to ensure that biometric feedback is clinically meaningful but also useful in real-world diabetes care, future work should test the design of user-centered feedback systems that integrate multiple data streams, optimize feedback frequency, and allow customization to individual needs.

## Figures and Tables

**Fig. 1.**
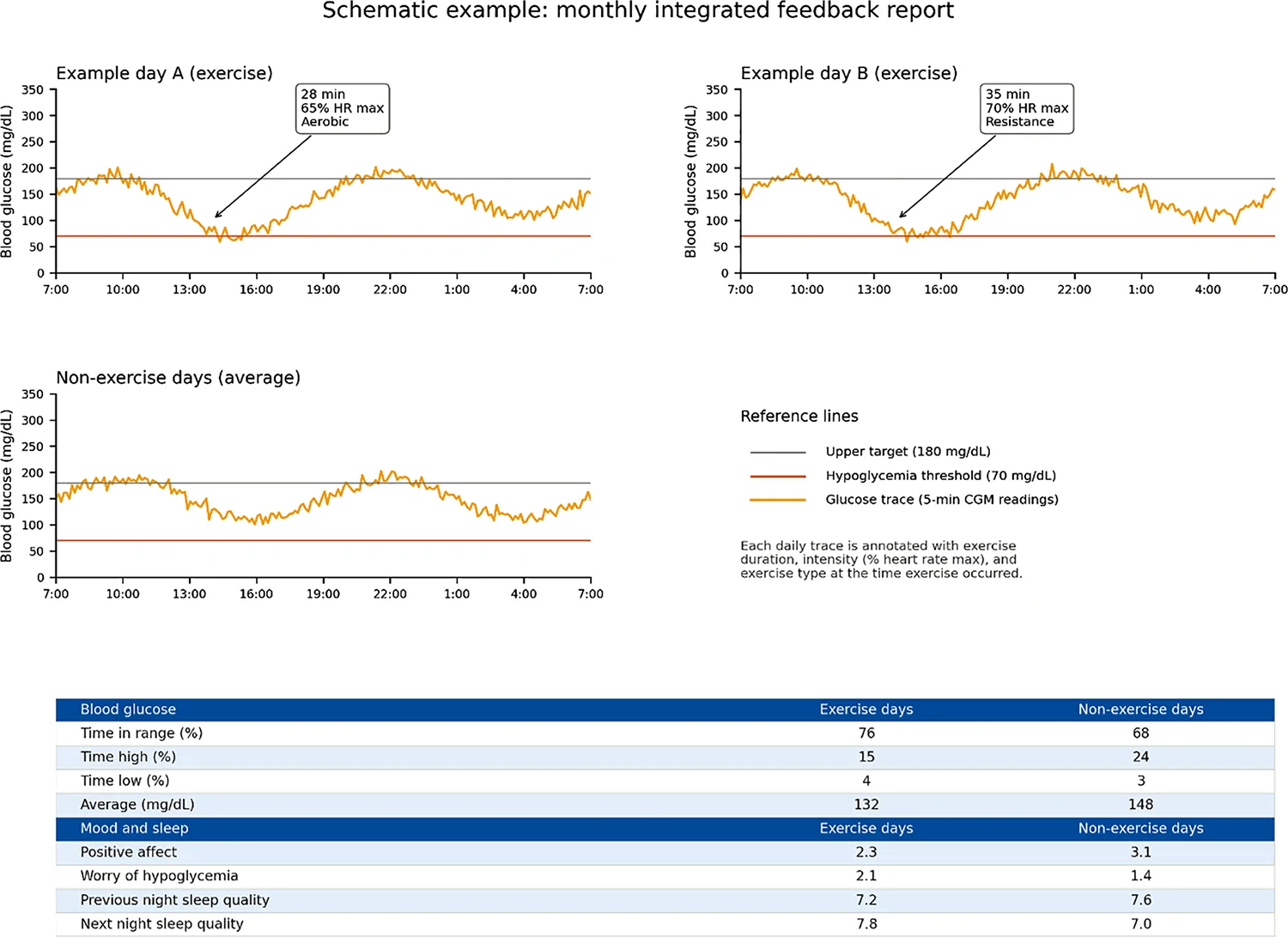
Illustrative schematic with representative example values of the monthly feedback report.

**Table 1:** Baseline participants’ clinical and demographic characteristics.

Demographics (n = 17)	M (SD) or %	Standard clinical recommendation
Sex (F)	52.9%	
Age (years)	43.4 (13.5)	
Race/Ethnicity	100% non-Hispanic White	
Income annual	5.9% 〈 $50,000; 35.3% $50,000–$79,999; 58.8% 〉 $80,000	
T1D duration (years)	23.1 (15.2)	
T1D therapy modality	53% automated insulin delivery (closed-loop insulin pump); 35% sensor-augmented insulin pump (manually operated pump); 12% multiple daily injections	
BMI (kg/m2)	29.5 (5.6)	18.5 to 24.9
HbA1c (%)	7.0 (1.2)	< 7.0

**Table 2-a:** Effects of exercise (days with exercise vs. without exercise) on *Hourly* Glucose measures after adjusting for time, weekday, age, duration of diabetes, BMI, and HbA1c: Time window from 18:00 in exercise day to 06:00 in next day.

Hourly Glucose Average (mg/dL)			
	Coefficient	StdErr	P-value
**Day with Exercise**	**−3.13**	**0.96**	**.0011**
Age	0.06	0.46	.8960
Diabetes Duration	−0.79	0.45	.0763
BMI	0.07	1.34	.9604
HbA1c	29.23	6.11	<.0001
Hourly Percent of Glucose in Range (%)			
	Coefficient	StdErr	P-value
**Day with Exercise**	**3.36**	**0.70**	**<.0001**
Age	0.10	0.26	.6980
Diabetes Duration	0.47	0.25	.0641
BMI	−0.45	0.75	.5490
HbA1c	−15.05	3.43	<.0001
Hourly Percent of Glucose Above 250 mg/dL (%)			
	Coefficient	StdErr	P-value
**Day with Exercise**	**−2.16**	**0.44**	**<.0001**
Age	0.15	0.19	.4392
Diabetes Duration	−0.40	0.19	.0318
BMI	−0.14	0.56	.8019
HbA1c	8.97	2.55	.0004
Hourly Percent of Glucose Below 70 mg/dL (%)			
	Coefficient	StdErr	P-value
**Day with Exercise**	**0.81**	**0.26**	**.0016**
Age	−0.02	0.05	.7135
Diabetes Duration	−0.02	0.05	.6636
BMI	0.24	0.14	.0986
HbA1c	−1.89	0.65	.0037

Note. The coefficients were obtained from Generalized Linear Mixed Model predicting CGM measures in each hour with random intercept. Each model includes time (hour) and weekdays, but the coefficients (i.e., 12 coefficients for 13 levels of hour & 6 coefficients for 7 weekdays) of hours are not presented in Table 2.

**Table 2-b:** Effects of exercise (days with exercise vs. without exercise) on the *Hourly* risk of hyperglycemia (Glucose ≥ 250 mg/dL) and hypoglycemia (Glucose≤70 mg/dL) *in 5 min* after adjusting for time, weekday, age, duration of diabetes, BMI, and HbA1c: Time window from 18:00 in exercise day to 06:00 in next day.

Hyperglycemia (≥ 250 mg/dL)			
	Odds Ratio	95% CI	P-value
Day with Exercise			**<.0001**
Exercise Day	**0.68**	**[0.58, 0.78]**	
No Exercise Day	Ref		
Weekdays			**<.0001**
Monday	Ref		
Tuesday	1.13	[0.91, 1.40]	
Wednesday	1.13	[0.91, 1.41]	
Thursday	1.11	[0.89, 1.37]	
Friday	1.42	[1.15, 1.75]	
Saturday	1.48	[1.20, 1.83]	
Sunday	0.97	[0.78, 1.21]	
Age	0.97	[0.78, 1.02]	.2448
Diabetes Duration	0.96	[0.91, 1.01]	.1237
BMI	1.04	[0.90, 1.22]	.5532
HbA1c	**3.89**	**[1.93, 7.82]**	**.0011**
Hypoglycemia (≤ 70 mg/dL)			
	Odds Ratio	95% CI	P-value
Day with Exercise			**.0567**
Exercise Day	**0.85**	**[0.71, 1.00]**	
No Exercise Day	Ref		
Weekdays			0.2577
Monday	Ref		
Tuesday	0.98	[0.75, 1.28]	
Wednesday	0.96	[0.73, 1.25]	
Thursday	1.26	[0.98, 1.63]	
Friday	1.06	[0.81, 1.38]	
Saturday	1.13	[0.87, 1.48]	
Sunday	0.96	[0.73, 1.25]	
Age	0.98	[0.94, 1.01]	.1539
Diabetes Duration	1.01	[0.98, 1.05]	.4118
BMI	1.08	[0.98, 1.18]	.1018
HbA1c	**0.46**	**[0.29, 0.71]**	**.0018**

Note. The coefficients were obtained from Logistic Regression predicting the likelihood of hyperglycemia (Over 250 mg/dL) in each 5 min with random intercept.

**Table 3:** Effects of Exercise (Post Exercise Periods: 30 min, 1 hr, 2 hrs, and Later) and Weekdays on Glucose Level *in 5 min* After Adjusting for Time, Age, Duration of Diabetes, BMI, and HbA1c: Time Window from 18:00 in Exercise Day to 06:00 in Next Day.

Glucose (mg/dL) in 5 min			
	Coefficient	StdErr	P-value
Periods After Exercise			
Within 30 mins	−3.93	0.72	<.0001
30 mins to 1 hr	−4.50	0.76	<.0001
1 hr to 2 hrs	−4.84	0.59	<.0001
Later 2 hrs	Ref		
Weekdays			
Monday	Ref		
Tuesday	1.49	0.45	.0010
Wednesday	1.58	0.46	.0005
Thursday	1.32	0.46	.0037
Friday	5.52	0.46	<.0001
Saturday	7.22	0.46	<.0001
Sunday	2.63	0.46	<.0001
Age	0.08	0.47	.8604
Diabetes Duration	−0.75	0.46	.0985
BMI	0.19	1.37	.8869
HbA1c	29.02	6.24	<.0001

Note. The coefficients were obtained from Generalized Linear Mixed Model predicting glucose level in each 5 min with random intercept.

**Table 4:** Effects of exercise (post exercise periods: 30 min, 1hr, 2 hrs, and Later) and weekdays on the risk of hyperglycemia (glucose ≥ 250 mg/dL) *in 5 min* after adjusting for time, age, duration of diabetes, BMI, and HbA1c: Time Window from 18:00 in exercise day to 06:00 in next day.

Hyperglycemia (≥ 250 mg/dL)			
	Odds Ratio	95% CI	P-value
Periods After Exercise			**<.0001**
Within 30 mins	**0.78**	**[0.69, 0.88]**	
30 mins to 1 hr	**0.73**	**[0.64, 0.83]**	
1 hr to 2 hrs	**0.57**	**[0.52, 0.64]**	
Later 2 hrs	Ref		
Weekdays			**<.0001**
Monday	Ref		
Tuesday	**1.17**	**[1.09, 1.26]**	
Wednesday	1.06	[0.98, 1.14]	
Thursday	**1.19**	**[1.11, 1.28]**	
Friday	**1.55**	**[1.44, 1.66]**	
Saturday	**1.61**	**[1.50, 1.72]**	
Sunday	0.94	[0.88, 1.02]	
Age	0.96	[0.89, 1.03]	.1953
Diabetes Duration	0.97	[0.90, 1.04]	.3483
BMI	1.08	[0.88, 1.32]	.4326
HbA1c	**4.96**	**[1.97, 12.48]**	**.0024**
Hypoglycemia (≤ 70 mg/dL)			
	Odds Ratio	95% CI	P-value
Periods After Exercise			**.4880**
Within 30 mins	**0.95**	**[0.80, 1.13]**	
30 mins to 1 hr	**1.03**	**[0.86, 1.23]**	
1 hr to 2 hrs	**0.90**	**[0.78, 1.04]**	
Later 2 hrs	Ref		
Weekdays			**<.0001**
Monday	Ref		
Tuesday	**1.16**	**[1.03, 1.30]**	
Wednesday	1.13	[1.01, 1.27]	
Thursday	**1.71**	**[1.54, 1.91]**	
Friday	**1.54**	**[1.38, 1.72]**	
Saturday	**1.39**	**[1.24, 1.55]**	
Sunday	1.07	[0.95, 1.21]	
Age	0.97	[0.93, 1.02]	.2188
Diabetes Duration	1.02	[0.97, 1.06]	.4496
BMI	1.08	[0.95, 1.22]	.2419
HbA1c	**0.42**	**[0.23, 0.76]**	**.0077**

Note. The coefficients were obtained from Logistic Regression predicting the likelihood of hyperglycemia (Over 250 mg/dL) in each 5 min with random intercept.

## Implications and applications

From a human factors perspective, findings from this study illustrate how rich and complex continuous biometric data from CGM can be analyzed and presented to enhance user understanding and support everyday decision-making around health behaviors. These results highlight the analytic feasibility of developing an integrated biometric feedback system to generate interpretable, clinically relevant information. The integrated system could link information from multiple data streams (e.g., CGM, exercise, potentially sleep or diet) into a single, interpretable dashboard that minimizes information overload or technological burden for users. By identifying clinically meaningful patterns in overnight glucose stability as well as high-risk time windows (e.g., weekends), such CGM-linked applications could help users anticipate changes in glycemic risk events, plan or adjust insulin or meals, and sustain motivation for exercise. This approach aligns well with human factors principles of usability, effective information integration, and workload reduction.

## Impact statement

This study lays the groundwork for use of an integrated feedback system that connects biometric data to everyday health behaviors among adults with T1D. Integrating human factors methods into the design of diabetes technologies can expand the role of CGM from a safety monitoring device to a proactive behavioral support tool. By applying usability and information integration principles to CGM-exercise feedback, this work contributes to a growing evidence base for digital tools that not only deliver accurate health information but also optimize comprehension, reduce anxiety about hypoglycemia and increase adherence to exercise. This study helps bridge clinical, behavioral, and human factors perspectives to advance the development of a clinically safe, low-burden system that empowers individuals with T1D to engage in sustainable diabetes self-management.
